# Evaluation of Cerebral Volume Changes in Patients with Tremor Treated by MRgFUS Thalamotomy

**DOI:** 10.3390/life13010016

**Published:** 2022-12-21

**Authors:** Federico Bruno, Emanuele Tommasino, Alessia Catalucci, Cristina Pastorelli, Francesco Borea, Giulia Caldarelli, Mattia Bellini, Pierfrancesco Badini, Sara Mancini, Chiara Santobuono, Saverio Martino, Valeria Pagliei, Guglielmo Manco, Davide Cerone, Francesca Pistoia, Pierpaolo Palumbo, Francesco Arrigoni, Ernesto Di Cesare, Carmine Marini, Antonio Barile, Alessandra Splendiani, Carlo Masciocchi

**Affiliations:** 1 Emergency Radiology, San Salvatore Hospital, 67100 L’Aquila, Italy; 2 Italian Society of Medical and Intervention Radiology (SIRM), SIRM Foundation, 20122 Milan, Italy; 3 Department of Biotechnological and Applied Clinical Sciences, University of L’Aquila, 67100 L’Aquila, Italy; 4 Neuroradiology and Interventional Radiology, San Salvatore Hospital, 67100 L’Aquila, Italy; 5 Mediaire GmbH, 10965 Berlin, Germany; 6 Neurology, San Salvatore Hospital, 67100 L’Aquila, Italy; 7 Department of Diagnostic Imaging, Area of Cardiovascular and Interventional Imaging, Abruzzo Health Unit 1, 67100 L’Aquila, Italy; 8 Department of Life, Health and Environmental Sciences, University of L’Aquila, 67100 L’Aquila, Italy

**Keywords:** tremor, MRgFUS thalamotomy, brain volumes, MRI

## Abstract

The purpose of the study is to quantify volumetric variations of cortical and subcortical brain structures after Vim ablation using MRgFUS, and correlate them with the patients’ clinical features and treatment outcomes. For this pilot retrospective study we enrolled 31 patients with a mean age of 70.86 years who were eligible for unilateral Vim thalamotomy. Clinical evaluation included tremor severity assessment using the FTM scale and cognitive assessment using the MoCA score. MRI data were acquired with a 3T scanner, using a dedicated 32-channel coil and acquiring a volumetric sequence of T1 3D IR FSPGR (BRAVO), before treatment and one year after MRgFUS thalamotomy. Image processing and volume data extraction were conducted with dedicated software. A volumetric analysis showed a significant reduction (*p* < 0.05) of the left thalamus 1 year after the treatment in patients with ET. Other significant results were found on the same side in the other nuclei of the basal ganglia and in the cerebellar cortex. In confronting the two groups (ET, PD), no significant differences were found in terms of age, FTM, MoCA scores, or brain volumes. Similarly, no significant correlations were found between the FTM and MoCA scores and the brain volumes before the treatment.

## 1. Introduction

Tremor is defined as an involuntary rhythmic movement involving a succession of contractions and the relaxation of muscles. It is the main symptom of various pathologies such as Essential Tremor (ET) and Parkinson’s disease (PD) [1]. The pathogenetic mechanisms underlying tremor change according to the pathology. Postmortem studies identified cerebellar abnormalities in individuals with ET, but the real pathogenesis cannot be declared with certainty [2]. Parkinsonian tremor is generated from impulses arising within the brain due to the degeneration of brain neurons that produce dopamine, a neurotransmitter with inhibitory functions on the basal ganglia [3]. The loss of function of intracerebral pathways involves the basal ganglia, thalamus, and cerebral cortex. It is believed that the rhythmic activity of a central generator, identified in the intermediate ventral nucleus (Vim) of the thalamus, contributes to the genesis of tremor [4]. Tremor can be treated pharmacologically, but many of the drugs used have variable efficacy and carry an increased risk of side effects such as dyskinesia and motor fluctuations. Currently, surgical treatment can be considered a valid option in patients with disabling tremor who are not responsive to pharmacological treatments [5]. Transcranial thalamotomy of Vim using magnetic resonance guided high-intensity focused ultrasound (tc-MRgFUS) is a new minimally invasive thermal ablation procedure that does not require surgical access and represents a safe and effective treatment option for tremor in patients with ET and PD [6,7].

Thanks to the most recent software that allows for the segmentation and automated cortical reconstruction of high-resolution MR images, it is possible to obtain data about the volumetric brain variations in patients with tremor (PD or ET) to better delineate the morphological and functional changes related to the specific pathology and relate them to the patient’s outcome. Numerous studies have used imaging techniques to demonstrate the presence of structural and functional brain alterations in patients with TE and PD [8,9,10]. Some previous reports in the literature also explored the possible prognostic value of imaging finding, using standard and advanced imaging techniques, in patients submitted to pharmacological, surgical, and less invasive treatments for tremor [11,12,13,14,15].

In one of our previous experiences, the correlation between volumetric parameters of brain structures (white matter, gray matter, and cerebrospinal fluid) as predictors of procedural outcome in patients undergoing MRgFUS thalamotomy was investigated [16]. At present, there is no longitudinal evaluation of the changes in brain volumes of patients treated with MRgFUS thalamotomy. In this context, our study is proposed as a pilot study to quantify volumetric variations of the different brain structures after MRgFUS ablation in patients with ET and PD and correlate them with the patient’s clinical and prognostic outcome.

## 2. Materials and Methods

### 2.1. Study Population

We conducted a retrospective study including 31 patients, 7 women and 24 men, with a mean age of 70.86 ± 6.2 years (range 53–87). Patients were affected by Parkinson’s disease (n = 16) and disabling essential tremor (n = 15) refractory to drug therapy; patients included in the study underwent unilateral MRgFUS Vim thalamotomy treatment at the Radiology department of the “San Salvatore” Hospital of L’Aquila. The patients were all right-handed and were treated for a right tremor (left Vim ablation).

### 2.2. Clinical Evaluation

From electronic medical records, we retrieved clinical data, which were examined by two neurologists with expertise in movement disorder (DC, FP). According to our selection protocol, the neurological examination includes a pre-treatment clinical evaluation based on a collection of demographic data, a detailed clinical history and a quantitative assessment of tremor with the Fahn-Tolosa-Marin Tremor Rating Scale. Cognitive assessment was also conducted using the MocA test, a widely used screening test for detecting mild cognitive impairment. The same clinical tests were administered, according to our clinical protocol, after 1 months, 6 months, and 1 year after treatment. For the purposes of the study, clinical data at 1-year follow-up were recorded, including post-procedural complications. All participants included in the study gave their written informed consent before the procedure. Patients with incomplete clinical records were excluded from the study.

### 2.3. MRI Evaluation and Volumetric/Statistical Analysis

All MRI data were acquired with a clinical 3T scanner (MR750w, GE Healthcare, Chicago, IL, USA), using a dedicated 32-channel coil. The MRI standardized protocol included the sequences FLAIR (slice 3–0.3, TR 11,000, freq FOV 24, phase FOV 0.8), GRE (slice 3–0.3, TR 960, freq FOV 26, phase FOV 0.75), SWI (slice 2 mm, freq FOV 24, phase FOV 0.85) and DWI (slice 3–0.3, TR 10,550, freq FOV 26, phase FOV 0.8) on axial planes, T2 (slice 3.0–0.3, TR 7854, freq FOV 26, phase FOV 0,8) on axial and coronal planes, and a volumetric sequence T1 3D IR FSPGR (BRAVO) (slice 1 mm, TR 8,5, freq FOV 25.6, phase FOV 0.8) with multiplanar reconstructions.

**Figure 1:**
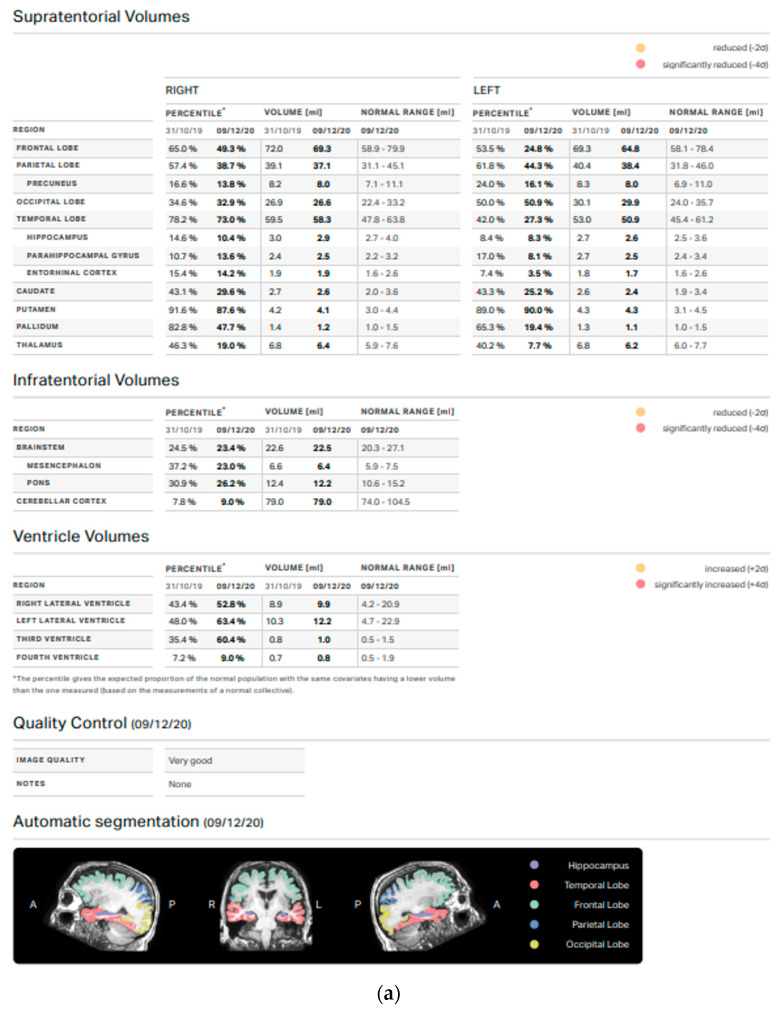

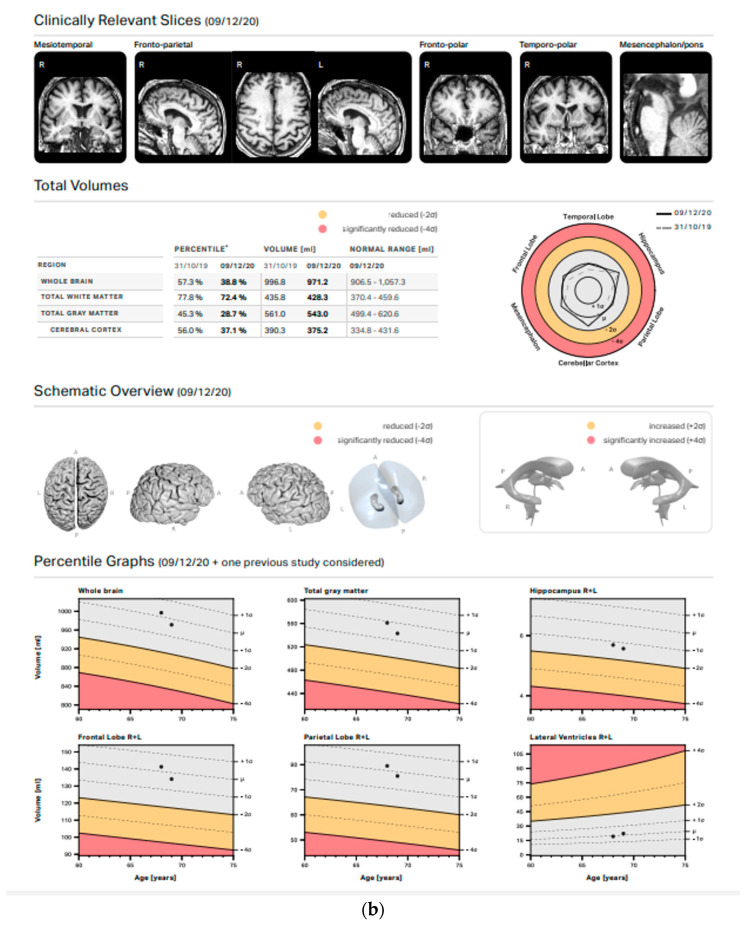
(**a**) Screen showing the interface for using the software dedicated to volumetric analysis, displaying detailed data of the supratentorial, infratentorial, and ventricular volumes, (**b**) Screen showing the interface for using the software dedicated to volumetric analysis, displaying clinically relevant slices and a schematic overview with percentile graphs.

Following the institutional follow-up protocol, all participants received an MRI scan before treatment and one year after the MRgFUS thalamotomy. Standard MRI scan sequences were analyzed before including a patient in the study; patients with structural brain abnormalities involving gray or white matter were excluded from volumetric analysis. Image processing and volume data extraction were conducted with Mdbrain software (Mediaire, Germany), a set of automated tools for morphometric analysis of the brain from structural MRI data. The 3D image files of BRAVO T1-weighted sequences (slice 1 mm, TR 8.5, freq FOV 25.6, phase FOV 0.8) were transferred to a workstation for morphometric analysis. Mdbrain automatically segmented the brain into several cortical and subcortical regions of interest. The volume values are calculated using a fully convolutional neural network architecture based on the widely used U-NET architecture (Ronneberger et al.: *U-net: Convolutional networks for biomedical image segmentation*, 2015). Using a normative database of several thousand individuals aged 18–92 years, mdbrain can also derive normal values based on age, sex and intracranial volume. The analysis was inspected for accuracy, and any geometric inaccuracies were corrected. This procedure allowed for the obtaining of the volumes (in mL) of different brain structures before and after the MRgFUS thalamotomy treatment from each patient. Particular attention was paid to total white matter, total gray matter, the cerebral cortex, the frontal lobe, the parietal lobe, the occipital lobe, the temporal lobe, the nuclei of the base, the thalamus, and the cerebellar cortex (Figure 1). Only patients with volumes in the normal range were included.

### 2.4. Statistical Analysis

Data analyses were performed using MedCalc (Version 20.121). Qualitative variables were summarized as frequency and proportions. Values of continuous variables were tested for normal distribution using a Shapiro–Wilk test and reported as means and standard deviations (SD) or medians and interquartile ranges (IQR) according to their distribution. Differences of quantitative values (age, FTM, MoCA, brain volume) between groups were compared using the Wilcoxon test or the Student’s *t*-test according to their distribution. The variance of FTM scores at the different follow-up times was evaluated using ANOVA for repeated measures. Point biserial correlation was applied to evaluate correlation between continuous and binomial variables. A correlational analysis of continuous variables was performed by a Spearman correlation test.

## 3. Results

### 3.1. Patients Population

**Figure 2:**
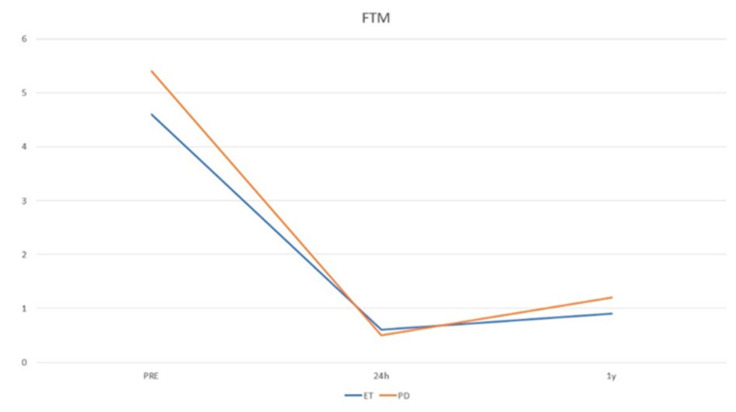
Trend of tremor intensity according to the FTM scale.

**Figure 3:**
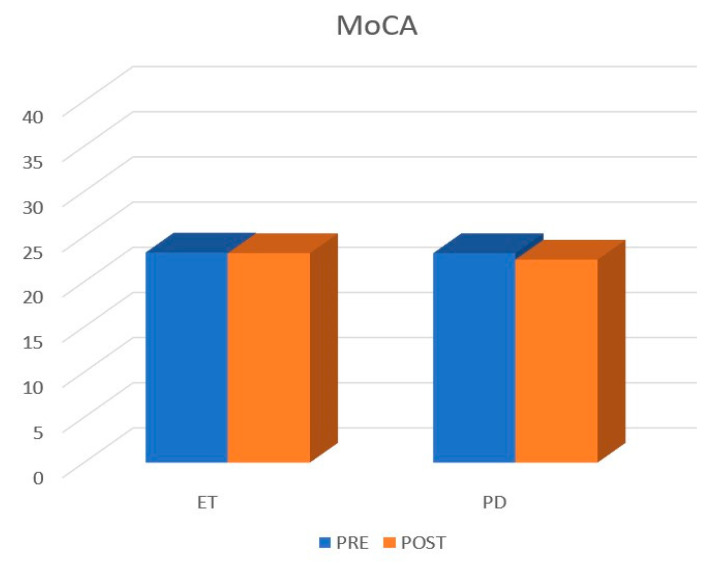
Cognitive assessment according to the MoCA score.

The study included 31 patients, 7 women and 24 men, with a mean age of 70.86 years (range 53–87). Baseline population characteristics are reported in Table 1. Patients affected by Parkinson’s disease (n = 16) showed a mean age of 68.4 years (range 51–85), a pre-FTM of 5.43 ± 2.39, a post-FTM 24 h of 0.50 ± 0.89 (*p* < 0.05), a post-FTM 1Y of 1.23 ± 1.36 (*p* < 0.05), a pre-MoCA of 23.18 ± 4.63, and a post-MoCA of 22.46 ± 7.17. A total of 5 out 16 patients presented with tremor relapse after the treatment. Patients affected by disabling essential tremor (n = 15) showed a mean age of 68.86 years (range 54–83), a pre-FTM of 4.60 ± 2.09, a post-FTM 24 h of 0.60 ± 0.79 (*p* < 0.05), a post-FTM 1Y of 0.93 ± 0.95 (*p* < 0.05), a pre-MoCA of 23.25 ± 5.09, and a post-MoCA of 23.81 ± 4.83. A total of 3 out 15 presented tremor relapse after the treatment (Figure 2 and Figure 3). No post-procedural complications were observed at the 1-year follow-up.

### 3.2. Volumetric Analysis

Brain volumes are reported in Table 2 and Table 3, where we report only the most significant changes.

A volumetric analysis showed a significant reduction (*p* < 0.05) of the left thalamus 1 year after the treatment in patients with ET. In particular, the mean value at the time of the procedure was 7.64 ± 0.70, while at the MRI follow-up, it was 7.38 ± 0.82. Other significant results were found on the same side in the other nuclei of the basal ganglia. In particular, the mean values at the time of the procedure of the left putamen and left pallidum were 4.32 ± 0.47 and 1.36 ± 0.17, respectively, while at the MRI follow-up, they were 4.22 ± 0.43 and 1.28 ± 0.17, respectively. Finally, the cerebellar cortex showed a total and significantly reduced volume one year after the MRgFUS (84.99 ± 14.69 vs. 91.11 ± 12.69) (Table 2).

The rest of the cerebral area did not show any significant difference before and after the treatment.

In patients with PD instead, no significant differences were found in the brain before or after the treatment (Table 3).

In confronting the two groups (ET & PD), before and after treatments, no significant differences were found in terms of age, FTM, and MoCA scores. Brain volumes were also the same, with the mere difference of the right caudate nucleus, which was bigger in patients with ET (3.58 vs. 3.17, *p* < 0.05).

No significant correlations were found between the FTM and MoCA scores and the brain volumes before the treatment. Similarly, the correlation between the brain volumes before the treatment and the tremor relapse rate did not reach an acceptable level of significance.

## 4. Discussion

In patients with tremor, numerous studies have explored the use of advanced imaging sequences to demonstrate the presence of structural and functional brain alterations and to evaluate the diagnostic value of brain volumes, both to differentiate the various pathological pictures (mainly essential tremor and PD tremor), and to correlate the imaging evidence with the clinical symptoms [8,9,17,18,19,20].

Despite the variability in outcome and study consistency, several studies revealed the presence of brain volume changes in many cortical and subcortical regions, with evidence of volume reduction in the basal ganglia, as well as volume increase in the frontal, temporal lobe and the anterior cingulate cortex [21,22,23].

Recently, with the advent of and innovative minimally invasive methods of functional neurosurgery, such as MRgFUS, the field has opened up to the exploration of their possible prognostic values. Regarding MRgFUS Vim thalamotomy, Tommasino et al. demonstrated the correlation between volumetric parameters of brain structures (white matter, gray matter, and cerebrospinal fluid) as predictors of procedural outcome [16]. However, to the best of our knowledge, there are no published studies evaluating the longitudinal changes in brain volumes of patients treated with MRgFUS thalamotomy. The preliminary results of the present study demonstrated a significant volume reduction at the level of the treated thalamus and ipsilateral basal ganglia structures. It would be logical to assume that these findings related to the targeted basal ganglia structures (thalamus, putamen and pallidum) may be due to the treatment. Nevertheless, further explanations would be necessary in order to understand whether volumetric changes relating to directly untreated areas, such as the cerebellum, can be attributed to effects induced by the treatment itself (atrophy) or are due to the normal pathological atrophy. VBM studies showed that compared to healthy patients, the brain volumes of the ET group were significantly smaller in many brain regions, including the caudate body, the middle temporal pole, the precuneus and the superior temporal gyrus while, compared to PD, the thalamus and the middle temporal gyrus were smaller [6]. Benito-Léon et al. also found a positive correlation between cortical atrophy in several brain regions involved in movement control and increased motor unit synchronization (more severe tremor). The most involved cortical regions affected by volume reduction were the left orbitofrontal cortex, the left isthmus of the cingulate gyrus, the right paracentral lobule, the right lingual gyrus, as well as reduced left supramarginal gyrus, right isthmus of the cingulate gyrus, left thalamus, and left amygdala volumes [19].

As a result, the differences observed are more likely linked to the cerebellar pathophysiology in ET rather than to the treatment itself.

Interestingly, regarding the cerebellar volume, several studies seem to show a significant decrease in ET, which is consistent with the data collected by our analysis with the automated software that compared them with a database of a reference population (8,9). In these studies, ET patients particularly demonstrated reduced volumes of lobules I-II, left Crus II, left VIIB, and an increased volume of right X when compared with the HC group. Cerebellar atrophy in ET patients is in agreement with several reports, which showed significant changes in the cerebellar architecture and loss of the Purkinje cells in patients with ET. In addition, Choi et al. also found that ET patients with head tremor (HT) presented more cerebellar atrophy that ET without it. This may be due to the heterogeneity of the ET patients or to a localized atrophy in patients without HT and a widespread one in ET with HT [8,10].

Although there are studies in the literature that attest to the absence of particular differences between the brain volumes of patients with PD compared to the relative control groups, no data is available about the changes in brain volumes before and after MRgFUS treatment. Our work seems to confirm a substantial stability in the brain volumes of patients with Parkinson’s over time (after 1 year) and following MRgFUS thalamotomy.

It is also interesting to note that moderate changes in brain volume (in particular at the level of the caudate, pallidum, putamen and thalamus) have been noted in patients with PD treated with DBS. Different pathological effects of DBS due to electrode insertion have been taken into account, such as the development of astrogliosis, inflammatory reaction, the formation of collagen scars and axonal damage in chronic stimulation. In addition, patients that underwent DBS appeared to have a decrease in microglia activation. Although speculative, these changes in caudate, pallidum, putamen and thalamus may represent a form of Wallerian degeneration [24]. There have also been also several studies that aimed at predicting the response to DBS based on the preoperative volume changes. For instance, Younce et al. demonstrated that preoperative larger ventricles and smaller thalamic volumes correlated with poorer motor response to STN DBS. Similarly, the risk of the freezing of gait and of falls has been correlated with reduced volumes of the putamen and the left postcentral gyrus, respectively [25].

Furthermore, a statistically significant difference was found regarding the right caudate nucleus, whose volume is approximately 12% greater in patients with ET than in PD. No results have been found in the literature to confirm this finding.

Generally speaking, although ET and PD have a different pathological substrate, there are few stable biomarkers available on a neuroanatomical level for distinguishing between these two conditions. A study conducted on a small sized sample, however, would seem to highlight some significant volumetric variations: in particular in patients with ET, volumes of the thalamus and the temporal cortex are lower than in PD; at the same time the cerebellar and frontal cortex volumes would be lower in patients with PD than in those with TE. These differences may be explained by the effects of compensation or self-reorganizations, due perhaps in part to the enhanced thalamocortical sensorimotor interaction and the head-eye position readjustment in the thalamus and in the temporal cortex, respectively.

Another study by Prodoehl et al. [26] showed, on a study population of 20 PD patients, a reduced activation through BOLD sequences of the prefrontal cortex and of the basal nuclei (in particular the pale globe) in patients with non-tremorgenic disease compared to tremor-dominant patients, without, however, any evident differences in white and gray matter volumes assessed with VBM.

Interestingly, brain volume differences also imply brain regions larger in these two populations. For instance, ET patients compared to healthy controls showed a significant increase in volume in the right caudate nucleus, pallidum, amygdala, bilateral putamen and nucleus accumbens [17]. In our results, we found that before the treatment, patients with ET had a bigger caudate nucleus compared to PD patients. The literature and our results may also explain why ET patients showed significant volume reduction at the level of the caudate after the treatment while this did not happen in PD patients, since the pathogenic model may be different between the two groups. To explain volume changes in the caudate, putamen and pallidus, Prasad et al. postulated that these changes were likely to occur secondary to Purkinje cell death, as we mentioned beforehand. Indeed, the lower GABAergic transmission secondary to Purkinje cell degeneration may trigger a reduction of the inhibitory output from the cerebellar deep nuclei to the ventral lateral posterior nucleus (VLp). This could further induce a higher excitatory output from the VLp to the motor cortex, with a consequent increased cortical output to the pons, and hyperactivity of the cerebellar network as a final result. Moreover, the increased excitatory input which the motor cortex receives from the VLp may lead to a sequential increase in the excitatory output from the motor cortex to the basal ganglia [17]. Increased excitation of the striatum can potentially increase the inhibitory output of the GPi, subsequently reducing the inhibitory output of the GPi to the ventral lateral anterior nucleus (VLa), and thereby increasing the excitatory output to the motor cortex. These changes may lead to hyperactivity of the basal ganglia network and therefore hypertrophy. MRgFUS, inducing a blockage of the proposed network, may therefore be involved in the volume changes in ET after the treatment, since a reduction of the hyperactivity of the basal ganglia network determines a reduction of the volume of the nuclei.

The same hypothesis is corroborated by the results of other studies showing an increase in BOLD activation in the dorsolateral prefrontal cortex in TDPD patients compared to healthy controls, suggesting an increase in metabolism at the level of the thalamo-motor projections [26,27].

From a clinical point of view, the alterations found at the level of the basal nuclei and cerebellum were not associated with the presence of motor complications or cognitive alterations. The possible effects of the treatment on cognitive functions have been studied in numerous previous publications, and also with regard to the possible bilateral applications of the treatment. As already confirmed, unilateral Vim thalamotomy with MRgFUS does not cause changes in cognitive performance, even in long-term follow-up [28,29].

A note of caution is due in the analysis of cerebellar cortical volumes, as while the treatment is unilateral, our automated volume analysis did not separate the cerebellar cortex to the ipislateral/contralateral side of lesion, as it did for supratentorial structures. Nevertheless, we know that most cerebellar neural pathways, including the dentato-rubro-thalamic tract, are bilateral and decussating, and this could partially address this limitation [12,30,31].

Another important aspect concerns the identification of possible prognostic factors that could explain the onset of relapse after treatment. From a clinical point of view, Parkinsonian tremor is known to relapse more frequently than essential tremor, while gender, age or disease duration are not significant factors. Among the imaging findings, some studies suggest that the size of the thalamotomy lesion may be instrumental in determining the stability of treatment effects [11,12].

To the best of our knowledge, brain volumes had never been considered as a possible prognostic factor after treatment with MRgFUS. In our population, the volumetric differences of supra and subtentorial white and gray matter structures, and their post-treatment modifications, revealed no significant correlation with the trend in tremor intensity measured by the FTM scale.

## 5. Conclusions

This is the first study evaluating cerebral volume changes after MrgFUS thalamotomy intervention. In our experience we have found volumetric changes in patients with essential tremor but not in patients with Parkinson’s. Volumetric variations do not appear to be progrostic factors of the clinical effects of the treatment. Some limitations of our study need to be emphasized, in particular the relative limitation of the study population. The scarcity of studies with large populations present in the literature, together with the inhomogeneity of the methods of evaluation and calculation of brain volumes, makes a direct comparison of the results difficult, although there are significant elements in line with some current lines of thought. Further investigations on a larger sample would be necessary to better understand and contextualize this result and interpret its clinical significance.

## Figures and Tables

**Table 1:** Clinical and demographic baseline characteristics of the study population.

	PD	ET	*p*-Value
Age	68 ± 9.89	68 ± 9.79	0.12
Pre-FTM	5.43 ± 2.39	4.60 ± 2.09	0.23
Pre-MoCa	23.18 ± 4.63	23.25 ± 5.09	0.34

**Table 2:** Pre- and post-treatment brain volumes in ET patients.

Cerebral Area	Pre-Treatment Volume (mL)	Post-Treatment Volume (mL)	*p* Value
Total white matter	473.11 ± 53.47	471.96 ± 55.75	0.72
Total grey matter	654.50 ± 57.29	650.38 ± 62.31	0.27
Right thalamus	7.56 ± 0.66	7.50 ± 0.76	0.24
Left thalamus	7.64 ± 0.70	7.38 ± 0.87	0.001
Right Temporal lobe	65.68 ± 6.50	65.58 ± 7.51	0.84
Left Temporal lobe	61.32 ± 5.82	60.85 ± 6.52	0.37
Right Putamen	4.18 ± 4.86	4.14 ± 0.46	0.23
Left Putamen	4.32 ± 5.36	4.22 ± 0.43	0.001
Right Parietal lobe	75.37 ± 5.36	46.24 ± 4.53	0.33
Left Parietal lobe	46.73 ± 4.54	45.56 ± 46.70	0.33
Right Pallidus	1.35 ± 0.18	1.30 ± 0.17	0.06
Left Pallidus	1.36 ± 0.17	0.28 ± 0.17	0.004
Right Caudate	3.58 ± 0.55	3.63 ± 0.56	0.24
Left Caudate	3.36 ± 0.65	3.38 ± 0.68	0.53
Right Occipital lobe	32.625 ± 3.52	32.30 ± 4.31	0.47
Left Occipital lobe	34.12 ± 3.59	34.09 ± 4.65	0.95
Right Frontal lobe	32.62 ± 8.21	32.30 ± 9.02	0.47
Left Frontal lobe	34.12 ± 8.25	34.09 ± 9.11	0.95
Cerebral cortex	451.69 ± 42.36	451.93 ± 47.36	0.93
Cerebellar Cortex	91.11 ± 12.69	84.99 ± 14.69	0.02

**Table 3:** Pre- and post-treatment brain volumes in PD patients.

Cerebral Area	Pre-Treatment Volume (mL)	Post-Treatment Volume (mL)	*p* Value
Total white matter	500.36 ± 54.21	493.08 ± 55.21	0.07
Total grey matter	655.56 ± 69.43	651.23 ± 67.69	0.25
Right thalamus	7.67 ± 0.77	7.66 ± 0.71	0.83
Left thalamus	7.68 ± 0.80	7.58 ± 0.67	0.23
Right Temporal lobe	67.48 ± 8.60	67.00 ± 8.16	0.23
Left Temporal lobe	63.18 ± 8.43	62.59 ± 7.69	0.18
Right Putamen	4.03 ± 0.91	4.03 ± 0.68	1.00
Left Putamen	4.22 ± 0.70	4.18 ± 0.57	0.42
Right Parietal lobe	45.81 ± 4.84	45.63 ± 4.85	0.76
Left Parietal lobe	45.46 ± 5.54	45.91 ± 4.92	0.51
Right Pallidus	1.31 ± 0.27	1.29 ± 0.22	0.63
Left Pallidus	1.36 ± 0.27	1.35 ± 0.22	0.68
Right Caudate	3.17 ± 0.52	3.21 ± 0.61	0.63
Left Caudate	2.92 ± 0.55	2.95 ± 0.49	0.64
Right Occipital lobe	33.26 ± 3.88	33.41 ± 3.84	0.68
Left Occipital lobe	34.89 ± 3.16	34.61 ± 2.98	0.56
Right Frontal lobe	84.24 ± 11.88	84.70 ± 12.56	0.69
Left Frontal lobe	82.13 ± 12.54	82.75 ± 10.91	0.67
Cerebral cortex	456.48 ± 53.76	456.60 ± 52.65	0.97
Cerebellar Cortex	88.20 ± 13.83	86.61 ±16.35	0.42

## Data Availability

The data that support the findings of this study are available from the corresponding author, F.B., upon reasonable request.
